# A Case of Factitious Hyperglycemia in a Patient on Intravenous Ascorbic Acid

**DOI:** 10.1155/2018/7063137

**Published:** 2018-05-14

**Authors:** Eliza Sharma, Christine Resta, Patricia Park

**Affiliations:** ^1^Department of Internal Medicine, Maimonides Medical Center, Brooklyn, NY, USA; ^2^Division of Endocrinology, Maimonides Medical Center, Brooklyn, NY, USA

## Abstract

Finger stick blood glucose meters are widely used in outpatient and inpatient settings. However, various factors can affect accuracy of readings from these meters. Here we present a patient who had spurious glucose elevation on these meters while being on intravenous ascorbic acid.

## 1. Introduction

Finger stick blood glucose (FSBG) meters are widely used to measure blood glucose levels in outpatient and inpatient settings; however, various factors can affect accuracy of glucose readings from these meters [1–4]. Here we present a case of marked interference with FSBG readings due to intravenous ascorbic acid.

## 2. Case Report

**Figure 1:**
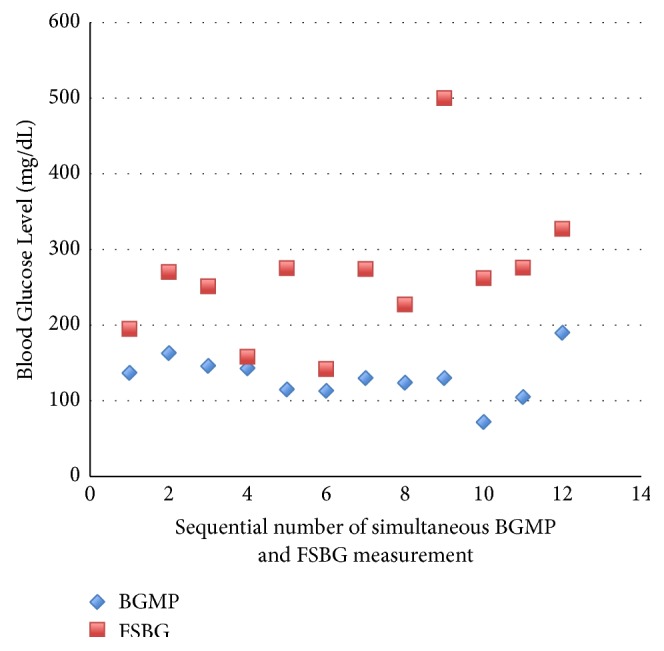
Discrepancy between simultaneous finger stick blood glucose (FSBG) and blood glucose in metabolic panel (BGMP).

An 81-year-old female underwent coronary artery bypass graft surgery and replacement of mitral valve, with postoperative course complicated by dehiscence of sternal wound requiring wound vacuum assisted closure, sacral decubitus ulcer, multiple episodes of sepsis, and respiratory failure. Her past medical history was significant for prediabetes and end stage renal disease on hemodialysis. The patient's HbA1C at presentation was 6%, and her blood glucose was well-controlled during the first 45 days of admission with only 2 units total of sliding scale insulin required. She was started on intravenous ascorbic acid 10000 mg every 2 days to aid in sternal wound healing. Soon afterward, the patient had multiple high FSBG levels over 200 mg/dL, and she was treated with a total of 9 units of insulin in 24 hours. The FSBG levels remained high over the next few days with readings above 250 mg/dL. The patient was started on daily 10 units of glargine insulin, which was increased to 25 units over the next few days. Of note, blood glucose in the metabolic panel (BGMP) ranged from 72 to 146 mg/dL, and a comparison of simultaneous FSBG and BGMP revealed a marked discrepancy of greater than 100 mg/dL between readings (Figure 1). These BGMP readings were obtained in the biochemistry laboratory of the hospital by the standard spectrophotometric technique. Given the suspicion for intravenous ascorbic acid causing falsely high FSBG readings in this individual without previous insulin requirements, it was discontinued. FSBG values decreased to normal. Fortunately, the patient did not have significant hypoglycemia while receiving the unnecessary insulin treatment.

## 3. Discussion

Measurement of serum glucose concentration by spectrophotometric technique using hexokinase method continues to be the gold standard and is used in clinical laboratories. However, monitoring of blood glucose using FSBG meters has greatly aided diabetes management. These FSBG meters make use of different enzymes including glucose oxidase, glucose dehydrogenase nicotinamide adenine dinucleotide (GDH-NAD), GDH flavin adenine dinucleotide (GDH-FAD), and GDH pyrroloquinolinequinone (GDH-PQQ). The enzymatic reaction of these enzymes with blood glucose generates an electrical current, the strength of which is proportional to the blood glucose level. FSBG meters relying on this analytic method, known as amperometric detection, are widely used in outpatient and inpatient settings. However, they are subject to varying degrees of interference by different factors including hematocrit, blood oxygenation, presence of other sugars like maltose, and so on [1–4]. Furthermore, the Manufacturer And User Facility Device Experience (MAUDE) database maintained by the United States Food and Drug Administration links a large number of adverse events to improper use of these glucose meters in patients [5].

Ascorbic acid acts as an antioxidant and a cofactor and is required for different biological processes including synthesis of collagen and norepinephrine [6, 7]. High dose ascorbic acid is a nutraceutical used mainly by alternative medicine practitioners for various indications. It has long been used to aid in wound healing [8–10]. The benefit of ascorbic acid in cancer patients has been debated but continues to draw interest and wide use [11–14]. Patients with severe sepsis have a significantly depleted level of ascorbic acid, and its early supplementation has been shown to prevent morbidity and mortality in these patients [15, 16]. This has led to an increasing use of intravenous vitamin C in critical care setting. Physicians should, however, be aware of the potential for high dose intravenous ascorbic acid to cause erroneously high readings on FSBG meters [17–19]. This spurious glucose elevation results from glucose oxidase and glucose dehydrogenase oxidizing ascorbic acid with generation of electrical current measured as glucose. The error arises when the meter reads ascorbic acid as glucose, as the two molecules have similar molecular weights. In our patient, falsely high FSBG led to potentially harmful interventions while, in some other reported cases, patients developed life-threatening hypoglycemia [19]. Physicians must, therefore, understand the limitations of blood glucose meters. Most laboratories measure serum glucose by spectrophotometric technique using the hexokinase method, which is specific for glucose and not affected by ascorbic acid. In this enzymatic method, hexokinase converts glucose to glucose-6-phosphate, which is then oxidized with concurrent reduction of nicotinamide adenine dinucleotide phosphate. The resulting increase in absorbance is measured. Furthermore, understanding the pharmacokinetics of ascorbic acid and waiting for at least 8–10 hours after intravenous ascorbic acid before reverting to FSBG meters to monitor blood glucose is preferred [20]. Blood glucose meters that utilize spectrophotometric methods are also available and may be used to avoid interferences in patients receiving intravenous ascorbic acid.
